# M2 Macrophages-Derived Exosomal miRNA-23a-3p Promotes the Progression of Oral Squamous Cell Carcinoma by Targeting PTEN

**DOI:** 10.3390/cimb45060314

**Published:** 2023-06-07

**Authors:** Jun Li, Yongjie Bao, Sisi Peng, Chao Jiang, Luying Zhu, Sihai Zou, Jie Xu, Yong Li

**Affiliations:** 1 College of Stomatology, Chongqing Medical University, Chongqing 401147, China; 2 Chongqing Key Laboratory for Oral Diseases and Biomedical Sciences, Chongqing 401147, China; 3 Chongqing Municipal Key Laboratory of Oral Biomedical Engineering of Higher Education, Chongqing 401147, China

**Keywords:** OSCC, M2 macrophages, exosome, MiR-23a-3p, PTEN

## Abstract

Exosomes from tumor cells and immune cells regulate the tumor microenvironment through the biomolecules or microRNAs (miRNAs) they carry. This research aims to investigate the role of miRNA in exosomes derived from tumor-associated macrophages (TAMs) in the progression of oral squamous cell carcinoma (OSCC). RT-qPCR and Western blotting assays were used to determine the expression of genes and proteins in OSCC cells. CCK-8, Scratch assay and invasion-related proteins were utilized to detect the malignant progression of tumor cells. High-throughput sequencing predicted differentially expressed miRNAs in exosomes secreted by M0 and M2 macrophages. Compared with exosomes from M0 macrophages, exosomes from M2 macrophages led to enhanced proliferation and invasion of OSCC cells and inhibited their apoptosis. High-throughput sequencing results show that miR-23a-3p is differentially expressed in exosomes from M0 and M2 macrophages. MiRNA target gene database predicts that phosphatase and tensin homolog (PTEN) are target genes of miR-23a-3p. Further studies revealed that transfection of miR-23a-3p mimics inhibited PTEN expression in vivo and in vitro and promoted the malignant progression of OSCC cells, which was reversed by miR-23a-3p inhibitors. MiR-23a-3p in exosomes derived from M2 macrophages promotes malignant progression of OSCC. PTEN is a potential intracellular target of miR-23a-3p. MiR-23a-3p, an M2 macrophage-associated exosome, is a promising target for the future treatment of OSCC.

## 1. Introduction

More than 90% of oral cancers are reported to be squamous cell carcinomas [1]. Oral squamous cell carcinoma is considered the most common maxillofacial malignancy, with a high rate of lymph node metastasis and a low five-year survival rate [2]. Currently, the treatment strategy combines surgery, radiotherapy, chemotherapy, and other methods, but achieving satisfactory results takes time [3]. Thus, it is necessary to find a more effective systemic therapy.

One of the critical components of stromal cells is tumor-associated macrophages, which are associated with poor prognoses [4]. Macrophages adopt different polarization states depending on the type of stimulus. Two central polarization states of the macrophage phenotype have been described: M1 macrophages and M2 macrophages [5]. M1 macrophages tend to promote inflammation and counteract tumors, whereas M2 macrophages frequently resist inflammation and contribute to tumors [6]. The macrophages in TME are primarily M1 polarized and play the anti-tumor role in the early stage of the tumor. Meanwhile, in the late stage of cancer, the macrophages in TME can change from M1 to M2 macrophages and participate in the progression of the tumor [7]. The promoting effect of M2 macrophages on the development of tumors has been confirmed in many reports, including gastric cancer and OSCC [8,9]. A study also shows that when the proportion of M2/M1 macrophage increases, it is often associated with tumor proliferation and unfavorable prognosis [10]. However, the mechanisms underlying the promotion of OSCC progression by M2 macrophages are still in need of further exploration.

In the TME, cells secrete soluble factors and two types of extracellular vesicles: microvesicles and exosomes. Exosomes with a diameter of 30–100 nm are membranous vesicles that cells secrete into the extracellular environment, which contain many proteins, lipids and genetic materials from parental cells [11]. This is one of the main communication pathways in TME and it transmits a variety of proteins, mRNAs and miRNAs between cells. As small non-coding RNA, miRNA regulate gene expression at the post-translational stage [12]. They can be used as inhibitors or enhancers of crucial signaling pathways and proteins, affecting various aspects of cancer biology [13].

Based on the evidence described above, we collected exosomes from M0 and M2 macrophages and treated tumor cells with them in order to better explore the role played by M2 macrophage exosomes in tumors. This study shows that miRNA-23a-3p is abundantly expressed in M2 macrophage exosomes and promotes malignant progression of tumor cells. PTEN is a target potential for miRNA-23a-3p. This study offers a new target for the prevention and treatment of OSCC.

## 2. Materials and Methods

### 2.1. Cell Culture and Transfection

Cal-27 (ATCC, Manassas, VA, USA) cells were cultured at 37 °C in an incubator with 5% CO2 using high sugar BMEM medium (Biological Industries, Kibbutz, Israel) containing fetal bovine serum (Biological Industries, Israel). Cal-27 are seeded into a 6-well plate when they are cultured to a density of 70–80%. Cal-27 cells were transfected with miR-23a-3p mimic using EntransterTM-R4000 (Engreen, Beijing, China) following the protocols of Ribobio (Guangzhou, China).

THP-1 (ATCC, USA) were cultured with RPMI-1640 medium (Biological Industries, Israel). When the density of THP-1 reached 70–80%, 100 ng/mL PMA (Sigma-Aldrich, St Louis, MS, USA) was added for 24 h to induce M0 macrophages. The medium was then changed and the stimulation of M0 macrophages was continued with 20 ng/mL IL-4 (Sigma-Aldrich, USA) for 24 h to induce the formation of M2 macrophages [14].

### 2.2. Isolation and Characterization of Exosomes

M0 and M2 macrophages were cultured in serum-free RPMI-1640 medium for 24 h. Exosomes in the cell supernatant were obtained via differential centrifugation and stored at −80 °C. After the exosomes were diluted using 1× PBS, VivaCell Biosciences and ZetaView PMX 110 (Particle Metrix, Meerbusch, Germany) were used to measure the size and concentration of the exosomes. The system was calibrated using 110 nm polystyrene particles. The temperature was 26.62 °C.

### 2.3. Reverse Transcription Polymerase Chain Reaction (RT-PCR)

Total intracellular RNA was extracted by RNAiso Plus kit (TaKaRa, Nogihigashi, Japan). RNA concentration was identified by NanoDrop 2000 spectrophotometer (Thermo, Waltham, MA, USA). cDNA was generated using SYBR Premix Ex Taq II (TaKaRa, Japan). TB Green (TaKaRa, Japan) and cDNA were mixed, and qPCR was performed for 40 cycles in Bio-Rad’s PCR system. The total reaction volume was 20 µL. GAPDH and U6 were used as internal references. Relative quantification of genes was performed using the 2^−ΔΔCt^ method. 

### 2.4. Western Blot

The treated cells were lysed with RIPA (Beyotime, Shanghai, China) containing protease inhibitors. The protein concentration was determined with the BCA protein quantification kit (Beyotime, China). After that, the protein was separated by SDS-PAGE gel electrophoresis and transferred to a PVDF membrane. PVDF membranes were closed with 5% skim milk powder for 2 h at room temperature, and the primary antibodies CD206 (1:1000; CST), CD163 (1:1000; Abcam), CD68 (1:1000; Abcam), CD86 (1:1000; Bioss), CD63 (1:500; Abcam), CD9 (1:500; Abcam), Caspase-3 (1:750; Bioss), Caspase-9 (1:750; Bioss), MMP-2 (1:1000; HUABIO), MMP-9 (1:1000; HUABIO), E-cadherin (1:1000; Abcam), Vimentin (1:1000; Abcam), and PTEN (1:1000; Abcam) were incubated at 4 °C overnight. After incubation of the secondary antibody for 1 h at room temperature, ECL reagent (Beyotime, Shanghai, China) was added and the bands were observed by Bio-Rad GelDoc 2000.

### 2.5. Cell Counting Kit-8 Assay

Cell Counting Kit-8 was used to test cell viability. The cells were seeded into a 96-well plate (3 × 10^3^/well) in the cell viability assay. After cell adhesion, the cells were stimulated with macrophage exosomes for 0, 24, 48, 72 h. Then, 10 µL CCK-8 solution was added to each well and the absorbance value was measured at 450 nm after incubation for 1 h. 

### 2.6. Wound Healing Assay

The wound healing assay was performed to measure cell motility. Treated cells were inoculated into 6-well plates at a density of 5 × 105/mL and incubated for 24 h. Then, parallel lines were drawn in the plate, and the serum-free medium was used instead of the complete medium. Wound closure was observed under a microscope at 0, 24 and 48 h. Image J software version 1.44 (National Institutes of Health, Bethesda, MA, USA) was used to measure the wound area.

### 2.7. miRNA Sequencing and Bioinformatics

The supernatants of M0 and M2 macrophages were collected and centrifuged to remove cells and debris. miRNA extraction, library preparation, and sequencing were performed at a commercial facility (Ribobio, Guangzhou, China). The Illumina HiSeq 2500 platform was chosen for sequencing. Data were collected using Illumina analysis software 2.2.68 version. The target genes of miR-23a-3p were predicted in TargetScan, miRDB, and miRTarBase databases.

### 2.8. Animal Studies

BALB/c nude mice (female, 4 weeks old) were purchased from Slekkinda Laboratory Animal Co., Ltd. (Shanghai, China). The mice lived in an environment with sufficient light, air, food, and water, and no pathogens. Treated or untreated Cal-27 cells (2 × 10^6^ cells in 0.1 mL PBS per mouse) were injected subcutaneously into the axillae of mice (n = 4). After one month, tumor-bearing mice were euthanized, and tumor tissue was removed and weighed. BALB/c nude mice were cultivated following the Guidelines for the Care and Use of Experimental Animals.

### 2.9. Tunel Staining

Paraffin sections were dewaxed with xylene and treated with 20 μg/mL proteinase K for 15–30 min. They were then washed 3 times with PBS. Immediately after, FITC-labeled TUNEL detection solution (Beyotime, Shanghai, China) was dropped on the sample, followed by incubation at 37 °C for 60 min in the dark. PBS wash was followed by dropwise addition of DAPI containing anti-fluorescence attenuation medium (Solarbio, Beijing, China), then the coverslip was fixed. FITC-labeled TUNEL-positive cells were observed at 488 nm excitation wavelength.

### 2.10. Immunohistochemistry

Immunohistochemical staining for Ki67 (1:200, Bioss) was performed. After conventional paraffin embedding and section, xenograft tumors were dewaxed with xylene, hydrated with ethanol, and sealed with 3% H_2_O_2_ for 10 min. After sections were closed with 10% goat serum, primary antibodies were incubated overnight at 4 °C. On the second day, a second antibody coupled with HRP was used for testing. Diaminobenzidine (DAB) was used as a chromogenic substrate. All slides were restained with hematoxylin. Images of tissue-stained sections were obtained using Olympus microscope (Olympus, Tokyo, Japan).

### 2.11. Statistical Analysis

All experiments were repeated three times. The data for each group were expressed as the mean ± standard deviation or the mean ± standard error of each group’s mean (S.E.M.). Differences between groups were compared using *t*-test or one-way ANOVA. Statistical analyses and graphs were performed using SPSS 18.0 (Chicago, IL, USA) and GraphPad Prism 8 (La Jolla, CA, USA). *p* < 0.05 was considered to be statistically significant.

## 3. Results

### 3.1. Extraction and Identification of M0 and M2 Exosomes

**Figure 1:**
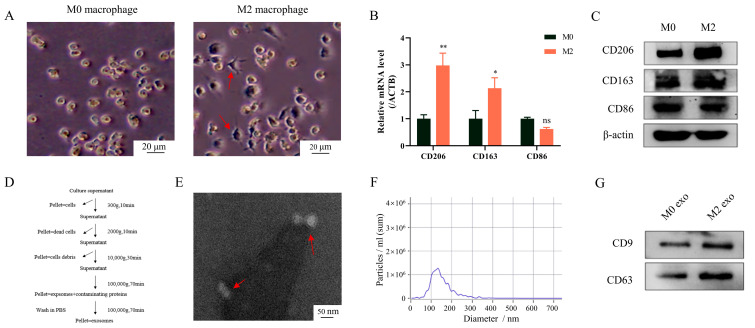
Extraction and identification of M0 and M2 exosomes. After 24 h of PMA (100 ng/mL) stimulation, THP-1 cell lines were treated with IL-4 (20 ng/mL) for 24 h. (**A**–**C**) Macrophage surface markers were detected. (**A**) Morphology of macrophages was observed under microscope. (**B**) RT-PCR analysis, (**C**) Western blot analysis. (**D**) Exosomes were obtained from cell supernatant via differential centrifugation. (**E**–**G**) Identification of exosomes: (**E**) Identification of exosome structure under an electron microscope (20,000×). (**F**) Detection of the size and number of exosomes via nanoparticle tracking analysis (dilution factor: 1:1000). (**G**) Detection of exosome markers with Western blot analysis. Data represent the mean ± standard errors of three separate experiments. * *p* < 0.05, ** *p* < 0.01, ns: not significant according to one-way ANOVA (versus M0 group).

After PMA and IL-4 stimulation, M0 and M2 macrophages were obtained. Under the microscope, M0 macrophages were observed to be round and M2 macrophages were observed to be spindle shaped (Figure 1A). Western blot and RT-PCR analysis were used to identify the surface markers of macrophages to demonstrate successful induction of M0 and M2 macrophages. The results showed that CD206 and CD163 were highly expressed in M2 macrophages, while CD86 (M1 macrophage marker) was not significantly different between M0 and M2 (Figure 1B,C). The exosomes in the supernatant were extracted via differential centrifugation (Figure 1D) and then identified. Transmission electron microscopy revealed that the vesicles isolated from the supernatant of macrophages have the characteristics of exosomes: double-layer membrane structure and disc shape (Figure 1E). As per the results displayed in the NTA detection, the diameter of the vesicles was mainly concentrated in 50–200 nm (Figure 1F). In addition, the exosome markers (CD9 and CD63) expression was observed via protein blot (Figure 1G). These findings illustrated that M0 and M2 macrophages were successfully induced, and their exosomes were extracted. 

### 3.2. M2 Macrophage Promotes Malignant Progression of OSCC

**Figure 2:**
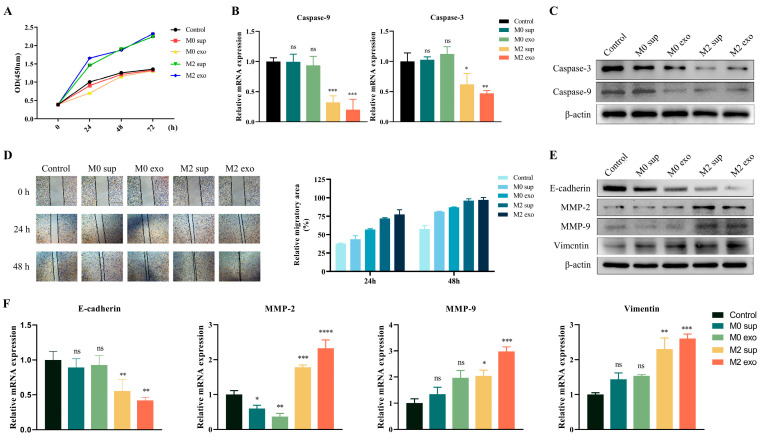
M2 macrophage exosomes promote the progression of OSCC cells (**A**) CCK-8 assays were performed to determine cell viability at 0, 24, 48, and 72 h after cell treatment. (**B**,**C**) Effect of M2 macrophage-derived exosomes on apoptosis-related cytokine in OSCC cells. (**B**) RT-PCR analysis. (**C**) Western blot analysis. (**D**) The migration activity of Cal-27 cells was measured via wound healing assay. The wound area was calculated at 0 h, 24 h, and 48 h after cell treatment. (**E**,**F**) The expression of EMT related protein in Cal-27 cells is affected by M2 macrophage-derived exosomes. (**E**) RT-PCR analysis. (**F**) Western blot analysis. * *p* < 0.05, ** *p* < 0.01, *** *p* < 0.001, **** *p* < 0.0001, ns: not significant by one-way ANOVA (versus control group).

Afterward, to probe the function of M2 macrophage exosomes on OSCC, CCK-8 was performed to detect the viability of tumor cells. As a result, compared with the control group, M0 sup group, and M0 exo group, the activity of Cal-27 cells treated with M2 macrophage supernatant and M2 macrophage exosomes increased significantly (Figure 2A). Additionally, Western blot and RT-PCR analysis observed that supernatants and exo of M2 macrophages significantly inhibited the expression of apoptosis-related proteins (Caspase-3 and Caspase-9) in tumor cells (Figure 2B,C). In contrast, there was no statistical difference between M0 sup, M0 exo, and the control group. These results suggest that exosomes of M2 macrophages inhibit the apoptosis of tumor cells and enhance their cell viability.

Subsequently, the promoting effect of M2 macrophages on tumor migration and invasion was also observed. M2 macrophage exosome treatment enhanced the migration ability of tumor cells (Figure 2D). The increased MMP-2, MMP-9 and waveform proteins, as well as the decreased E-calmodulin in tumor cells, tend to promote the process of epithelial–mesenchymal transition (EMT), thus favoring tumor expansion into adjacent tissues. The results showed that the expression levels of MMP-2, MMP-9 and Vimentin increased, and E-cadherin decreased in the cells treated with M2 sup and M2 exo. In contrast, the expressions of MMP-2, MMP9 and E-cadherin in the M0-sup and M0-exo groups were not significantly different from those in the control group (Figure 2E,F). The above conclusions indicate that M2 macrophage exosomes can enhance the proliferation and migration ability of Cal-27 cells. 

### 3.3. MiR-23a-3p Promotes Malignant Progression of OSCC Cells

**Figure 3:**
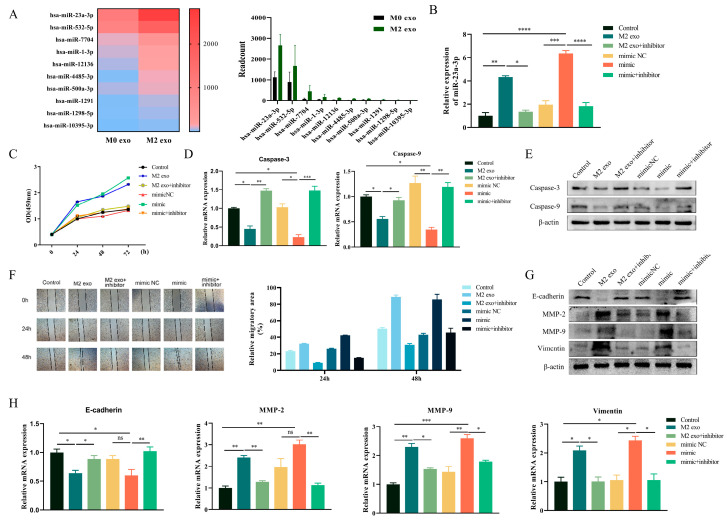
MiR-23a-3p promotes malignant progression of OSCC cells (**A**) In silico analysis of regulatory miRNAs related to M0 and M2 macrophage exosomes. Cal-27 was treated with M2 macrophage exosomes or miR-23a-3p mimics after stable transfection with miR-23a-3p inhibitor. (**B**) RT-PCR analysis of miR-23a-3p in Cal-27 cells after transfection. (**C**) CCK-8 assays were performed to determine cell viability at 0, 24, 48, and 72 h after cell treatment. (**D**,**E**) RT-PCR and Western blot were used to detect the levels of apoptosis-related factors. (**F**) The motility of Cal-27 cells was determined via wound healing assays. EMT-associated proteins in Cal-27 cells were detected via Western blot (**G**) and RT-PCR (**H**). * *p* < 0.05, ** *p* < 0.01, *** *p* < 0.001, **** *p* < 0.0001, ns: not significant according to one-way ANOVA.

To identify essential factors contributing to the malignant progression of OSCC cells in M2 exosomes, high-throughput sequencing was used to determine the differences in miRNA expression in M0 and M2 macrophage exosomes. Based on the high-throughput sequencing results, we obtained many miRNAs differentially expressed between M0 and M2 macrophages. Then, we screened the top ten miRNAs with statistical significance and the most considerable diversity. In the results, the content of miR-23a-3p in the exosomes of M2 macrophages was the highest. It was significantly higher than in the exosomes of M0 macrophages (Figure 3A). Next, we transfected Cal-27 cells with miR-23a-3p mimics (Figure 3B) to explore whether miR-23a-3p derived from M2 macrophage exosomes caused the malignant progression of Cal-27 cells described above. The results were consistent with our prediction that transfection of miR-23a-3p mimics caused Cal-27 cells to exhibit enhanced value-added capacity and inhibition of apoptosis (Figure 3C–E).

In line with this, high intracellular expression of miR-23a-3p also promoted the migration ability of Cal-27 cells (Figure 3F). Meanwhile, the expression of EMT-related proteins in tumor cells increased after transfection of miR-23a-3p, which facilitated its invasion (Figure 3G,H). These data indicate that MiR-23a-3p in exosomes secreted by M2-type macrophages can enter OSCC cells and enable OSCC cells to develop in a self-beneficial direction.

### 3.4. MiR-23a-3p Targets PTEN

**Figure 4:**
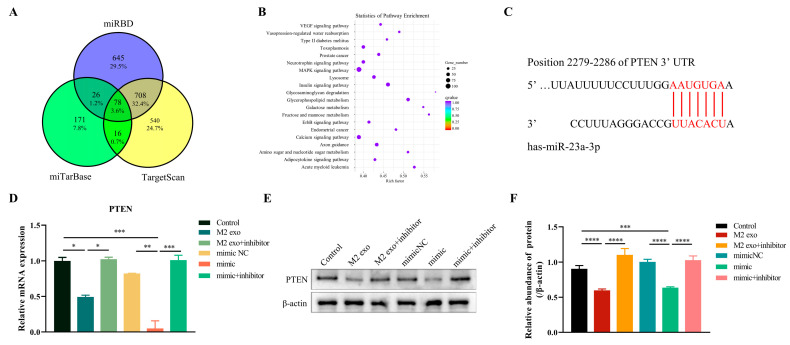
MiR-23a-3p targets PTEN (**A**) The three circles represent the target gene of miR-23a-3p in the three miRNA databases, and the middle part represents the intersection of the three datasets. (**B**) KEGG analysis of 78 target genes. (**C**) The predicted binding sites between miR-23a-3p and PTEN genes were predicted in the miRBD database. (**D**,**E**) RT-PCR and Western blot analysis of miR-23a-3p in Cal-27 cells. (**F**) Quantitative analysis. * *p* < 0.05, ** *p* < 0.01, *** *p* < 0.001, **** *p* < 0.0001 according to one-way ANOVA.

The TargetScan, miRDB, and miRTarBase databases were applied to predict the target genes of miR-23a-3p to reveal the mechanism of malignant progression of OSCC cells caused by miR-23a-3p derived from exosomes of M2 macrophages. Then, 78 target genes of miR-23a-3p were screened from three MiRNA databases, and pathway enrichment of 78 target genes was obtained (Figure 4A,B). Among the 78 target genes we obtained, PTEN is a common tumor suppressor gene known to play an essential regulatory role in the occurrence and development of tumors [15,16,17]. In addition, we detected a match between the sequence of miR-23a-3p and PTEN, further suggesting that PTEN is a potential target for miR-23a-3p (Figure 4C). Therefore, we further studied the effect of miR-23a-3p on the expression of PTEN in tumor cells. RT-PCR and Western blot results showed that PTEN expression in Cal-27 was significantly inhibited when M2 macrophage exosomes were used to stimulate tumor cells. The same results were obtained through transfection of tumor cells with miR-23a-3p mimics, but this was reversed by inhibitors of miR-23a-3p (Figure 4D–F).

### 3.5. MiR-23a-3p Promotes Tumor Growth In Vivo

**Figure 5:**
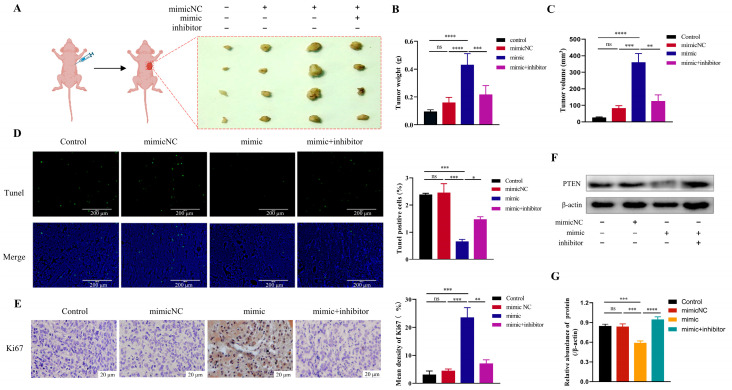
MiR-23a-3p promotes tumor growth in vivo. (**A**) The tumor. (**B**) Tumor weight. (**C**) Tumor volume. (**D**) Tunel-positive cells (green fluorescence) and their proportion. The nucleus is stained with DAPI (blue). (**E**) Immunohistochemical analyses of Ki67 in tumors. (**F**,**G**) Expression level of PTEN in tumor tissues. * *p* < 0.05, ** *p* < 0.01, *** *p* < 0.001, **** *p* < 0.0001, ns: not significant according to one-way ANOVA.

To further assess the effect of miR-23a-3p on tumors in vivo, we injected miR-23a-3p mimic-transfected Cal-27 cells subcutaneously into mice and observed the growth of tumors in vivo. The results showed a significant increase in tumor size and weight in the miR-23a-3p mimic group compared to the other groups (Figure 5A–C). In tumor specimens, the percentage of Tunel-positive cells was significantly lower in the miR-23a-3p transfected group, accompanied by an increase in Ki67-positive cells. This indicates that miR-23a-3p enables tumors to acquire greater proliferation ability (Figure 5D,E). In addition, consistent with the results of previous cellular experiments, miR-23a-3p also significantly inhibited the expression of PTEN in tumor tissues (Figure 5F,G).

## 4. Discussion

Macrophages aggregated in the TME are commonly referred to as TAMs. Many studies have shown that TAMs are effective promoters of tumor development and metastasis [18]. In particular, the tumor microenvironment will be altered during the polarization of M0 macrophages to M2-type macrophages. M2 macrophages tend to exert immunosuppressive effects to control inflammation and frequently promote tumor progression [6]. Recently, many studies have shown that exosomes with immunosuppressive activity are released mainly by M2 macrophages, promoting cancer progression and treatment resistance [19,20].

Exosomes have been studied in different diseases, and they are both pathogenic and protective. They play multiple roles in the microenvironment, reshaping the extracellular matrix (ECM) and mediating the transmission of signals and molecules between cells [21]. As cell-secreted vesicles, exosomes are readily absorbed by cells. Therefore, they have potential applications in cancer immunotherapy [22]. More and more studies have shown that exosomes can change their cell origin and disease state by transporting biologically active substances [23,24].

When we extracted exosomes from M0 and M2 macrophages to stimulate OSCC cells, we found that M2 macrophage-derived exosomes positively modulated OSCC progression, which manifested as enhanced cell proliferation and migration and inhibition of apoptosis. EMT is a process of epithelial dysfunction and increased motility that is critical in in tumor progression, metastasis, and drug resistance [25]. The upregulation of MMP-2, MMP-9 and Vimentin and the inhibition of E-cadherin in tumor cells will promote EMT and contribute to tumor invasion [26,27,28]. In subsequent studies, we also found that the exosomes of M2 macrophages enhanced the migration ability of tumor cells and promoted the TMT process by regulating the expression of MMP-2, MMP-9, Vimentin, and E-cadherin. These pieces of evidence suggest that exosomes of M2 macrophages benefit tumor growth and metastasis.

MiRNA and other non-coding RNAs are important regulatory components of exosomes, and they are taken up by surrounding cells during the exosomal cycle [12]. They can be used as inhibitors or enhancers of key signaling pathways and proteins, thus affecting different aspects of cancer biology [13]. MiRNAs not only play a role in transcriptional activation, epigenetic regulation, and translation inhibition, but have also been found in the mitochondria and nucleus [29]. We speculate that a specific miRNA is also responsible for the malignant progression of Cal-27 cells in the exosomes of M2 macrophages. Fortunately, we identified a highly differentially expressed miRNA in the exosomes of M0 and M2 macrophages via high-throughput sequencing: miRNA-23a-3pin. Then, we transfected Cal-27 cells with mimics of miRNA-23a-3p to verify the effect of miRNA-23a-3p on OSCC. Not surprisingly, we obtained the same results in Cal-27 cells transfected with miRNA-23a-3p as when stimulated with M2 macrophage exosomes. This suggests that miRNA-23a-3p in M2 macrophage exosomes can lead to the development of OSCC.

Immediately, to explore the mechanism of mirNA-23A-3p’s effect on OSCC cells, we analyzed the target genes of mirNA-23A-3p using the miRNA target gene database (targetscan, mirdb, and mirtarbase). PTEN mutation is one of the critical factors in human cancer development and is a known tumor suppressor gene [30,31]. Many studies have confirmed that the loss of PTEN can promote the development and metastasis of tumors, including breast cancer, testicular germ cell tumors, and cervical cancer [32,33]. In OSCC, the antitumor effect of PTEN has also been confirmed [34,35,36]. So, we boldly speculated that miRNA-23a-3p promoted the progress of OSCC by targeting the expression of PTEN in OSCC cells. We successfully observed a binding site between miRNA-23a-3p and PTEN and confirmed the targeting of PTEN by miRNA23a-3p at the cellular level. The tumor-promoting effect of miRNA-23a-3p achieved by inhibiting PTEN has also been observed in vivo.

## 5. Conclusions

**Figure 6:**
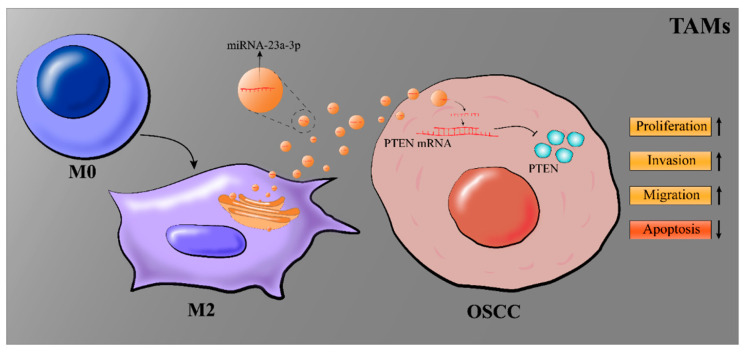
Graphical illustration of the effect of miRNA-23a-3p on OSCC cells.

In brief, the above findings suggest that M2 macrophage-derived exosomes promote OSCC cell proliferation, invasion, and migration and inhibit OSCC cell apoptosis by transferring miRNA-23a-3p into OSCC cells. Additionally, PTEN is a potential cellular target for miRNA-23a-3p to promote tumor development (Figure 6). This study may provide new biomarkers for the treatment of OSCC, but more work is still needed.

## Data Availability

Data supporting this study can be obtained according to the methods in this paper. Reasonable requests for data in the article can be obtained by contacting the corresponding author.
